# Maternal HtrA3 optimizes placental development to influence offspring birth weight and subsequent white fat gain in adulthood

**DOI:** 10.1038/s41598-017-04867-3

**Published:** 2017-07-04

**Authors:** Ying Li, Lois A. Salamonsen, Jonathan Hyett, Fabricio da Silva Costa, Guiying Nie

**Affiliations:** 1 Centre for Reproductive Health, Hudson Institute of Medical Research, Clayton, Victoria Australia; 2 Department of Molecular and Translational Science, Monash University, Clayton, Victoria Australia; 3 Department of Obstetrics and Gynaecology, Monash University, Clayton, Victoria Australia; 4 Central Clinical School, University of Sydney, Sydney, New South Wales Australia; 5 RPA Women and Babies, Royal Prince Alfred Hospital, Sydney, New South Wales Australia; 6 Monash Ultrasound for Women, Melbourne, Victoria Australia; 7 Department of Biochemistry and Molecular Biology, Monash University, Clayton, Victoria Australia

## Abstract

High temperature requirement factor A3 (HtrA3), a member of the HtrA protease family, is highly expressed in the developing placenta, including the maternal decidual cells in both mice and humans. In this study we deleted the HtrA3 gene in the mouse and crossed females carrying zero, one, or two HtrA3-expressing alleles with HtrA3^+/−^ males to investigate the role of maternal vs fetal HtrA3 in placentation. Although HtrA3^−/−^ mice were phenotypically normal and fertile, HtrA3 deletion in the mother resulted in intra-uterine growth restriction (IUGR). Disorganization of labyrinthine fetal capillaries was the major placental defect when HtrA3 was absent. The IUGR caused by maternal HtrA3 deletion, albeit being mild, significantly altered offspring growth trajectory long after birth. By 8 months of age, mice born to HtrA3-deficient mothers, independent of their own genotype, were significantly heavier and contained a larger mass of white fat. We further demonstrated that in women serum levels of HtrA3 during early pregnancy were significantly lower in IUGR pregnancies, establishing an association between lower HtrA3 levels and placental insufficiency in the human. This study thus revealed the importance of maternal HtrA3 in optimizing placental development and its long-term impact on the offspring well beyond *in utero* growth.

## Introduction

High-temperature requirement factor A (HtrA) proteins are a family of serine proteases with functional importance in regulating protein-protein interactions and protein folding stress^1^. To date, four mammalian HtrAs (HtrA1-4) have been identified^1–7^, and their dysregulation is associated with a number of diseases, including cancer, arthritis, neurodegenerative disorders, age-related macular degeneration, and pregnancy diseases^8–17^. In particular, HtrA1 and HtrA3 have been suggested as tumor suppressors, because they are down-regulated in a number of cancers and this reduction is suggested to promote tumorigenesis^18–22^. HtrA3 down-regulation in lung cancer is believed to occur because cigarette smoking induces methylation of the HtrA3 gene^8^. The reduced HtrA3 expression is further linked to diminished effectiveness of anti-cancer treatment of lung cancer^23^, and increased risk of postoperative recurrence of the tumor^24^.

HtrA3 was initially cloned, in both the mouse and human, from the developing placenta because of high HtrA3 expression^5, 6, 25, 26^. In both species, alternative splicing gives rises to two HtrA3 mRNA transcripts and two HtrA3 proteins isoforms, the long (HtrA3-L) and short (HtrA3-S) variants^5, 6^. HtrA3-L protein is comprised of five major domains, the signal peptide, IGF binding, Kazal inhibitor, trypsin-like serine protease and PDZ domains^5, 6^ (Supplementary Figure 1A). HtrA3-S lacks the C-terminal PDZ domain, but otherwise is identical to HtrA3-L^5, 6^ (Supplementary Figure 1A). HtrA3 gene structure and protein sequences are highly conserved between the mouse and human^5, 6^. While the mouse expresses the HtrA3-L isoform predominantly, both HtrA3 isoforms are produced comparably in the human^5, 6^. Both human HtrA3 isoforms are confirmed to be proteolytically active^27^. To date, it is unknown whether the two HtrA3 isoforms exert distinctive functions.

In the mouse, HtrA3 expression is markedly up-regulated in the uterus during placental development^6^. Specifically, the maternal decidual cells within the decidua basalis strongly express HtrA3, and the level is highest during early pregnancy when the placenta is actively developing^26^. In the human, HtrA3 is also abundantly expressed in the developing placenta, with the level being maximal during the first trimester of pregnancy^25^. Again, HtrA3 is highly expressed in maternal decidual cells during human placental development^25^. In women, HtrA3 is additionally expressed by a number of trophoblast subtypes including the villous syncytiotrophoblast, during the first trimester of pregnancy^25^. This placental HtrA3 is secreted into the maternal circulation with HtrA3 serum levels reflecting placental production, being highest in the first and lowest in the third trimester of pregnancy^12^.

*In vitro* studies indicate that HtrA3 negatively regulates trophoblast invasion during human placentation^28, 29^. Furthermore, serum levels of HtrA3 are altered during early pregnancy in women who subsequently develop preeclampsia in the third trimester^12, 30^. As defective placentation is a major cause of preeclampsia^31^, this data suggests an association between HtrA3 alteration and placental abnormalities. A recent study identified HtrA3 as a potential target of a prolactin family paralog in maternal decidual cells during mouse placental development^32^. However, to date, the functional importance of HtrA3 in placental development and function is unknown.

In the present study, we created an HtrA3 null mouse model and investigated the importance of HtrA3 in placental development. The HtrA3^−/−^ mice were fertile and phenotypically normal. As the mouse and human both develop a hemochorial placenta, requiring highly regulated participation of both fetal and maternal cells^33, 34^, and HtrA3 is highly expressed in maternal decidual cells during placentation^26^, we investigated the consequence of deleting the maternal vs fetal HtrA3 on placentation and fetal growth. Strikingly, HtrA3 deletion in the mother but not in the fetus, resulted in placental insufficiency and intra-uterine fetal growth restriction (IUGR). This IUGR, caused by HtrA3 deficiency in the mother, altered the growth trajectory of the offspring, independent of their genotype. To establish that the mouse data are relevant to the human, we also investigated the association between maternal serum levels of HtrA3 during placental development and IUGR in human pregnancies.

## Results

### Targeted disruption of the HtrA3 gene in the mouse

The structure of the mouse *HtrA3* gene (27760 bp, located on chromosome 5) is schematically illustrated in Supplementary Figure 1A. It contains 10 exons; alternative splicing leads to two HtrA3 mRNA transcripts (L-mRNA and S-mRNA), which translate into two protein isoforms (L-Protein, 459; S-Protein, 363aa)^5^. Exons 1–6 are transcribed into both HtrA3 mRNA transcripts, whereas exons 8, 9 and 10 are specific to the long and exon 7 is unique to the short HtrA3 mRNA transcript^5^ (Supplementary Figure 1A). Although exons 10 and 7 are the largest exons of the HtrA3 L-mRNA and S-mRNA respectively, both predominantly code for the 3′ untranslated region (Supplementary Figure 1A). In contrast, exon 1 is the largest coding exon of both HtrA3 L-mRNA and S-mRNA, containing the ATG translation start codon and coding for three protein domains (signal peptide, IGF binding and Kazal inhibitor domains) which are common to both HtrA3 protein isoforms (L-Protein and S-Protein)^5^ (Supplementary Figure 1A).

To remove both HtrA3 protein isoforms, we deleted the entire exon 1 and its flanking sequences with the targeting strategy shown in Supplementary Figure 1B. This deletion, further detailed in Supplementary Figure 1C, was predicted to prevent all HtrA3 transcription because an 880 bp regulatory sequence that precedes exon 1 was also removed.

**Figure 1:**
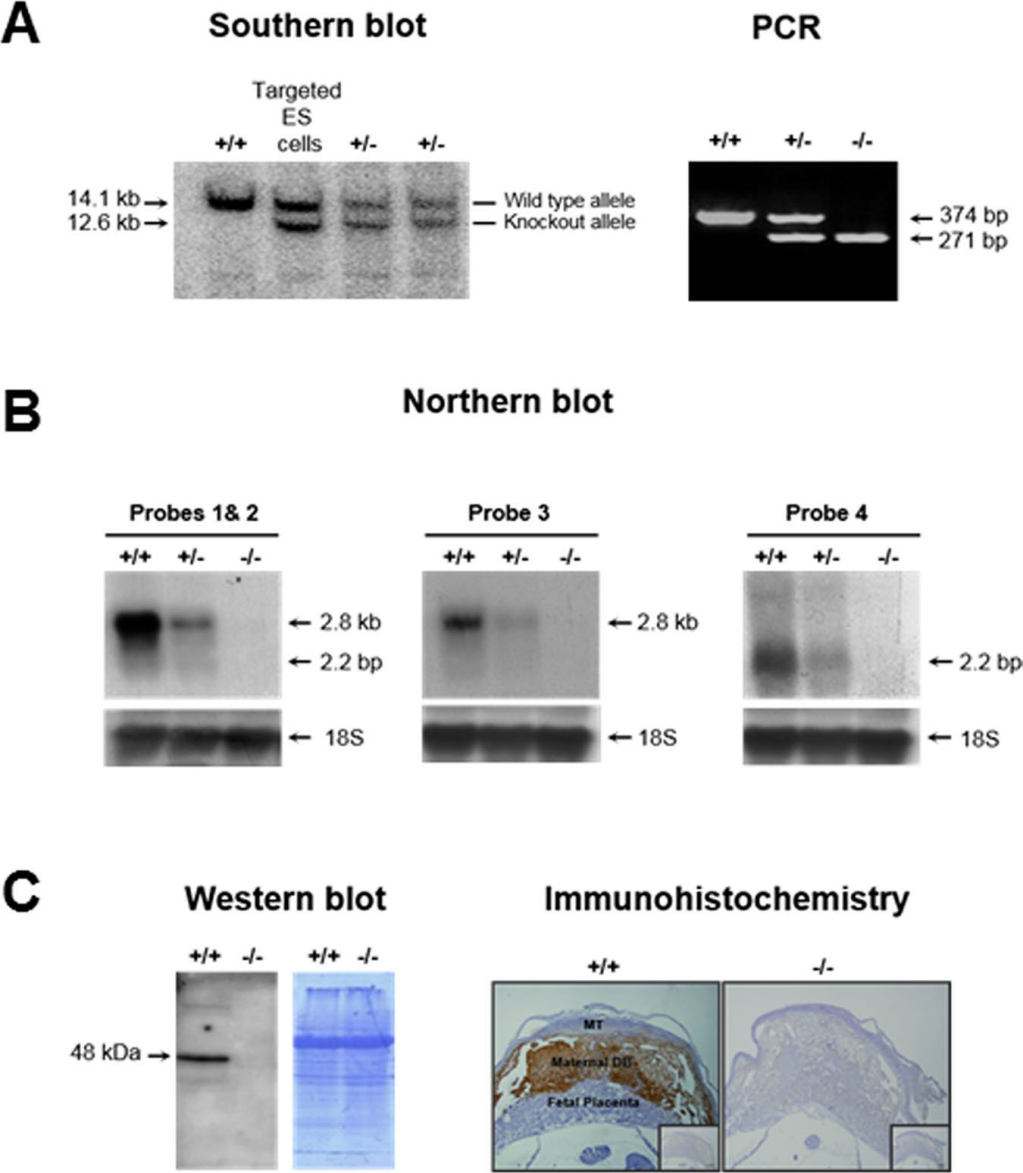
Confirmation of HtrA3 gene deletion in the mouse. (**A**) Southern blot analysis. Tail DNA from the wild type (+/+) and first generation heterozygous (+/−) mice, and genomic DNA from targeted ES cells, were digested with Sap1 and analyzed with the 3′ probe shown in Supplementary Fig. 1B. The band size of the wild type (14.1 kb) and the null (12.6 kb) alleles are shown. PCR analysis. Tail DNA from 3 representative HtrA3 genotypes of mice were analyzed by PCR using the 3 genotyping primers (F, R1 and R2) illustrated in Supplementary Fig.1C; the resulting products (374 and 271 bp) and band pattern were precisely as predicted. (**B**) Northern blot analysis. Total RNA from d10.5 implantation sites were analyzed by the 4 distinct probes shown in Supplementary Fig. 1(A). Probe 1, specific to exon 1 (deleted); probe 2, downstream of the deleted region and common to both HtrA3 mRNAs; probe 3, specific to HtrA3 L-mRNA (~2.8 kb); probe 4, unique to HtrA3 S-mRNA (~2.2 kb). Detection with probe 4 required a much longer exposure because of much lower expression of the HtrA3 S-mRNA. (**C**) Western blot analysis. Total protein lysates from d10.5 implantation sites were examined on two identical gels. Left panel, Western blot analysis; right panel, Coomassie blue staining showing equal loading. Immunohistochemistry. D10.5 implantation sites were analyzed, HtrA3 was immuno-stained (brown) predominantly in maternal decidua basalis of +/+ but not −/− mice; the inserts are negatives. MT, maternal uterine metrial triangle. An antibody detecting both HtrA3 protein isoforms were used for the protein analysis.

Southern blot analysis confirmed the correct targeting of ES cells and germline transmission of the deletion (Fig. 1A). Genomic DNA from ES cells and heterozygous mice born from chimeras was digested with SapI and probed with the 3′ probe illustrated in Supplementary Figure 1B. The wild type mouse showed a 14.1 kb band corresponding to the wild type allele, whereas the targeted ES cells and *HtrA3* ^+/−^ mice showed an additional band of 12.6 kb consistent with the null allele (Fig. 1A, Southern blot).

Intercrossing these *HtrA3* ^+/−^ mice produced three genotypes of mice, including *HtrA3* ^−/−^ homozygotes. Tail DNA from these mice was analyzed by PCR (Fig. 1A), using the scheme and primers illustrated in Supplementary Figure 1C. The band pattern and sizes of each genotype were exactly as expected (Fig. 1A and Supplementary 1C), validating the PCR strategy for genotyping.

Northern blot analysis confirmed a clear reduction in the *HtrA3* ^−/−^ heterozygotes, and a total deletion in the *HtrA3* ^−/−^ homozygotes, of both HtrA3 mRNA transcripts. Figure 1B shows the analysis of day (d)10.5 implantation sites, the tissue expressing high levels of HtrA3 in mice^26^. Four distinct probes, schematically shown in Supplementary Figure 1A, were employed to detect different parts of the HtrA3 mRNA: the deleted region (probe 1), sequence that is downstream of the deleted region but common to both HtrA3 mRNA transcripts (probe 2), and sequences specific to HtrA3 L-mRNA (probe 3) and S-mRNA (probe 4) variants. All four probes showed that the HtrA3 mRNA was reduced in *HtrA3* ^+/−^ and absent in *HtrA3* ^−/−^ mice (Fig. 1B).

Deletion of both HtrA3 protein isoforms was further confirmed by Western blot analysis of d10.5 implantation sites (Fig. 1C), using an antibody detecting both HtrA3 isoforms. No bands of HtrA3 protein were detected in the *HtrA3* ^−/−^ mice. Immunohistochemistry further corroborated this total deletion. In the wild type mice, HtrA3 protein was immuno-stained predominantly in the decidua basalis of d10.5 implantation sites (Fig. 1C), consistent with previous findings^26^. However, this staining was totally absent in the *HtrA3* ^−/−^ mice (Fig. 1C). These analyses confirmed total deletion of HtrA3 expression in the *HtrA3* ^−/−^ mice.

### Breeding analysis

The *HtrA3* ^−/−^ mice appeared normal, showing no obvious phenotypic abnormalities. To assess whether the HtrA3 deletion affects fertility, we set up all 9 possible crossings by mating females of three HtrA3 genotypes (+/+, +/− and −/−) with males of three HtrA3 genotypes (Table 1). All crossings produced viable pups in the expected Mendelian ratio, with no differences in litter size at birth or weaning (Table 1), indicating that HtrA3 deletion was not detrimental to female or male fertility.

**Table 1:** Breeding results from 9 different types of mating.

Mating (F × M)	n	Litter size at	
		Birth	Weaning
+/+ × +/+	7	6.9 ± 0.6	6.6 ± 0.7
+/+ × +/−	7	7.1 ± 0.6	6.7 ± 0.5
+/+ × −/−	6	6.2 ± 1.1	5.7 ± 0.8
+/− × +/+	3	6.0 ± 1.5	5.7 ± 1.5
+/− × +/−	7	7.7 ± 0.3	7.4 ± 0.2
+/− × −/−	6	6.5 ± 0.8	6.3 ± 0.9
−/− × +/+	3	7.3 ± 1.5	7.3 ± 1.5
−/− × +/−	6	6.0 ± 0.9	5.7 ± 1.0
−/− × −/−	8	7.4 ± 0.8	7.0 ± 0.8

**Figure 2:**
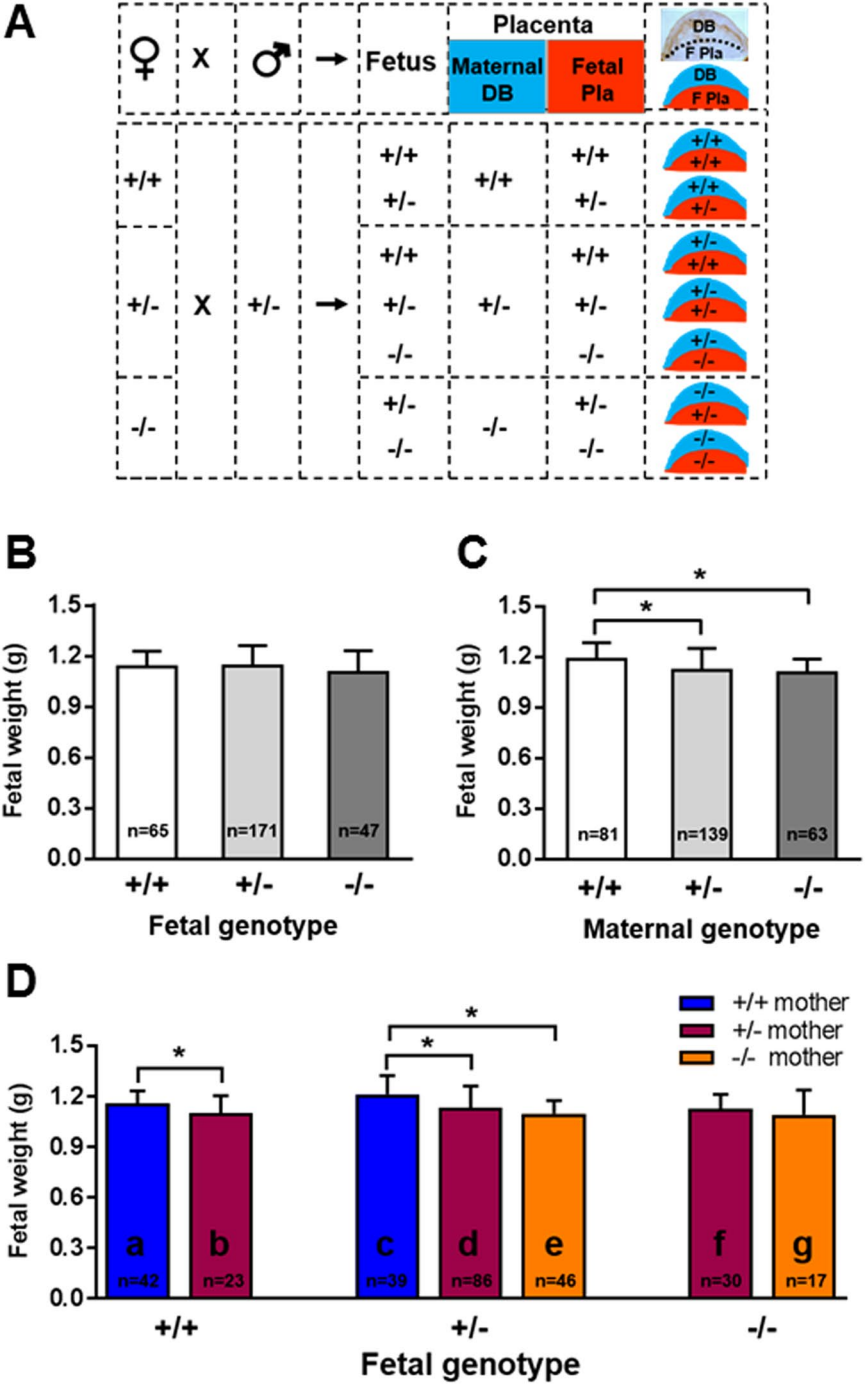
The impact of HtrA3 gene deletion in the mother versus fetus on E18 fetal weight. (**A**) The mating scheme to analyze the maternal and fetal contribution to the genomic makeup of the placenta. The top row illustrates that when a female (♀) is mated to a male (♂) mouse, the resulting fetus and placenta will be genetically related but not identical. The mouse placenta is comprised of maternal decidua basalis (DB) and fetal placenta (F Pla), genetically the maternal component will be identical to the mother, whereas the fetal component will be similar to the fetus. An actual image of a d10.5 placental section is shown at the far right top corner, displaying the maternal DB on the top (stained for desmin in brown) and the F Pla on the bottom. Immediately below it is a schematic drawing of the placenta, showing the maternal DB on the top in blue and F Pla on the bottom in red. The lower section of the figure shows the scheme for mating female mice of three HtrA3 genotypes (+/+, +/− or −/−) with males of HtrA3 heterozygous (+/−) to generate three HtrA3 genotypes (+/+, +/− or −/−) of fetuses but seven genotypes of placentas. Each placental type is schematically drawn to show the HtrA3 genotype of the maternal DB (top in blue) and the F Pla (bottom in red). (**B**–**D**) Fetal weight at E18. Data were analyzed according to the genotype of the fetus (**B**), the mother (**C**), and the fetus as well as the mother (**D**). The number of fetuses examined for each group is shown on the graph. The 7 distinctive groups in (**D**) are labeled as “a, b, c, d, e, f, g” respectively, and this labelling system is consistently applied to other figures. Data are expressed as mean ± SD. *p < 0.05.

As mouse HtrA3 is highly expressed in maternal decidual cells during placental development^26^, we next assessed whether HtrA3 deletion affects placental development and thus fetal growth. The mouse placenta is comprised of two major components, the maternal decidua basalis (DB) and the fetal placenta (F Pla) (Fig. 2A, top right corner)^26, 34^. The fetal placenta here refers to the labyrinth and the junctional zone. All these components are important for the development of an optimal placenta. During placentation, the maternal decidua regresses progressively whereas the fetal placenta enlarges dramatically^26, 35, 36^. Genetically, the maternal decidua is identical to the mother, whereas the fetal placenta is similar to the fetus. Therefore the fetal as well as the maternal genotype, which can be different from each other, jointly determine the placental genotype. As HtrA3 is expressed predominantly in the maternal decidua basalis (Fig. 1C)^26^, we employed the breeding strategy shown in Fig. 2A to determine the contribution of the maternal and fetal HtrA3 to placental development and fetal growth. Females of all three genotypes (HtrA3^+/+,+/−^ and ^−/−^) were crossed with heterozygous (HtrA3^+/−^) males to generate 3 possible genotypes of fetuses (HtrA3^+/+,+/−^ and ^−/−^) and 7 possible genotypes of placentas (Fig. 2A). The HtrA3 genotype of the maternal decidua basalis and the fetal placenta of each placental type is schematically illustrated in Fig. 2A.

These crossings produced viable fetuses in the expected Mendelian ratio (data not shown). There was no difference in embryonic day (E) 18 fetal weight when analyzed according to the fetal genotype (Fig. 2B). However, when analyzed according to the maternal genotype, E18 fetal weights differed: fetuses of HtrA3^+/−^ or HtrA3^−/−^ mothers were significantly lighter than those of HtrA3^+/+^ mothers (Fig. 2C), suggesting that HtrA3 deletion in the mother affected the fetal growth *in utero*. We next analyzed E18 fetal weight according to the genotype of the mother as well as the fetus, which separated them into 7 groups (Fig. 2D). The maternal genotype of HtrA3^+/+^ fetuses (Fig. 2D, left), could be HtrA3^+/+^ (Fig. 2D-a) or HtrA3^+/−^ (Fig. 2D-b). The mothers of HtrA3^+/−^ fetuses (Fig. 2D, middle), could be HtrA3^+/+^ (Fig. 2D-c), HtrA3^+/−^ (Fig. 2D-d) or HtrA3^−/−^ (Fig. 2D-e). The HtrA3^−/−^ fetuses (Fig. 2D, right), could come from HtrA3^+/−^ (Fig. 2D-f) or HtrA3^−/−^ (Fig. 2D-g) mothers. These 7 groups of fetuses were supported *in utero* by the 7 distinctive placentas respectively (Fig. 2D and A).

Within each fetal genotype, deletion of maternal HtrA3 reduced E18 weight (Fig. 2D). For the HtrA3^+/+^ fetuses (Fig. 2D, left), although all genetically similar (wild type), those from HtrA3^+/−^ mothers (Fig. 2D-b) had significantly lower E18 weight than fetuses from HtrA3^+/+^ mothers (Fig. 2D-a). For the HtrA3^+/−^ fetuses (Fig. 2D, middle), again all genetically similar, E18 weight was highest if the mother was HtrA3^+/+^ (Fig. 2D-c) and lowest if the mother was HtrA3^−/−^ (Fig. 2D-e); fetuses of HtrA3^+/−^ mothers (Fig. 2D-d) bridged the two extremes: their E18 weight was significantly lower than those of HtrA3^+/+^ mothers. A similar trend was apparent but not significant for the HtrA3^−/−^ fetuses (Fig. 2D, f-g). Since all differences were consistent between male and female fetuses: combined data is presented.

### Placental analysis

As fetal growth *in utero* depends on placental function, we analyzed the 7 genotypes of placentas that supported the above 7 groups of fetuses. The intact implantation sites containing the maternal decidua basalis and the fetal placenta were investigated at three developmental stages, d10.5, when the fetal placenta has just formed but is thin relative to the maternal decidua basalis; d13, when the fetal placenta has fully formed and the maternal decidua basalis is still present; and d16, when the fetal placenta has substantially expanded and the maternal decidua basalis has considerably regressed^26^.

H&E staining revealed no obvious differences in gross morphology among these placentas. Specific markers were thus employed to closely examine the decidua basalis and the fetal placenta respectively. The decidua basalis was analyzed by immuno-staining the stromal decidual cells for marker desmin^26^, and by lectin-DBA staining for uNK cells that constitute the most abundant immune cells in the decidua during placentation^26, 37^. The desmin staining pattern and NK cell presentation were similar among these placentas at all three developmental stages (data not shown). The decidual endothelium was examined by immuno-staining for Factor VIII^38^, and spiral arterial remodelling was analyzed by immno-staining for smooth muscle actin^38^. These analyses likewise revealed no clear differences, indicating that HtrA3 deletion did not affect the morphology of the maternal decidua basalis.

We also examined the junctional zone and the labyrinth of the fetal placenta. The gross architecture of these components was not different among these placentas. The glycogen cells, the major cell type of the junctional zone, were identified by staining with periodic acid–schiff (PAS) with and without digestion with α-amylase^38^, and immuno-staining for CX31^39^, but no major differences were apparent. Glycogen cells that have invaded into the maternal decidua basalis, immuno-stained for decorin^39^, also appeared to be similar (data not shown). These results thus suggest that HtrA3 deletion does not affect the glycogen cells within the junctional zone or their invasion into the maternal decidua.

The labyrinth is primarily responsible for nutrient and gas exchange between the mother and fetus. We closely examined the two major labyrinthine components, the maternal blood sinuses and fetal capillaries. The maternal blood sinuses, identified by staining the trophoblasts lining these spaces for alkaline phosphatase activity^40^, or for isolectin BS1 binding^41^, showed no clear differences.

**Figure 3:**
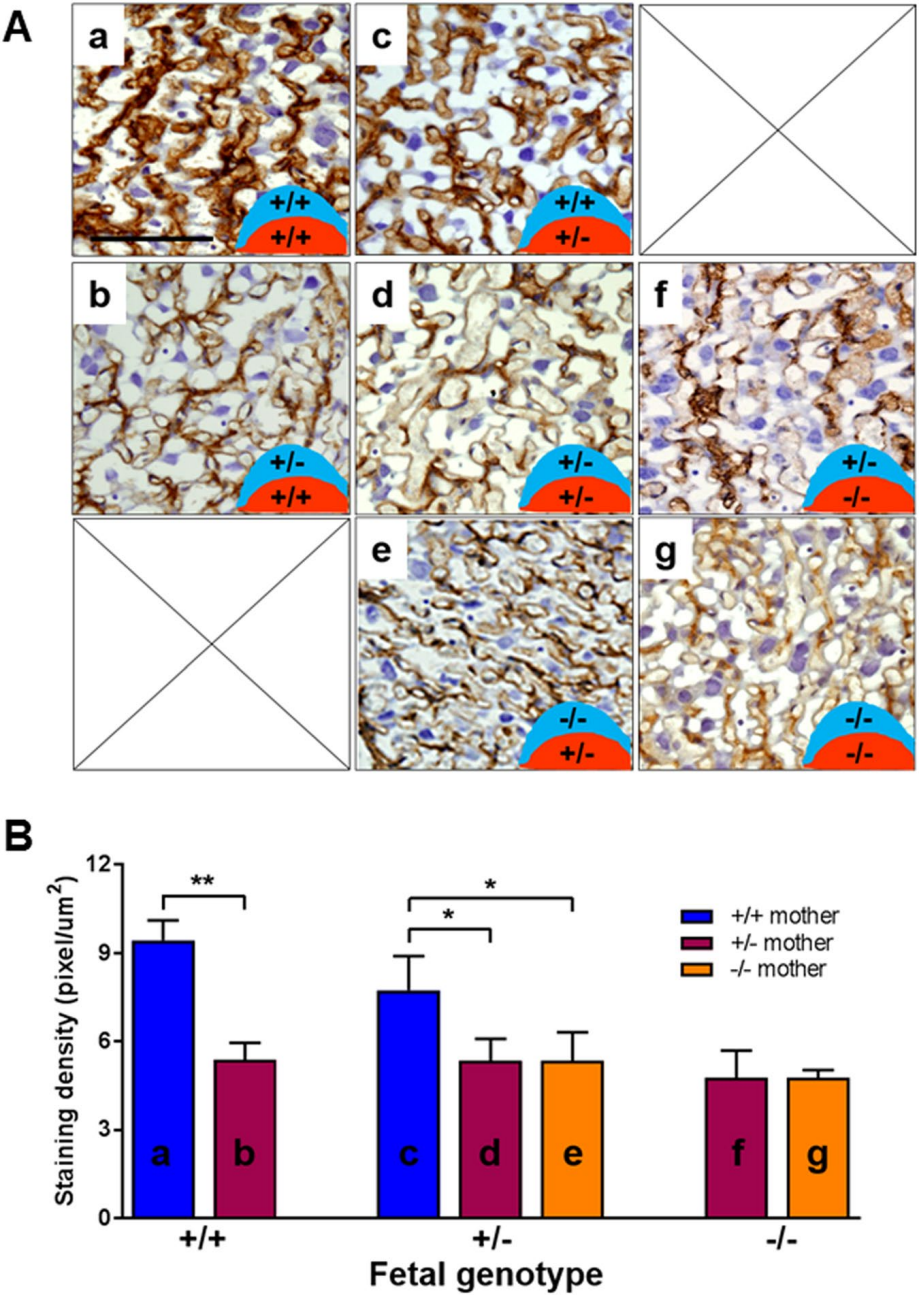
Analysis of fetal capillaries within the placental labyrinth at d16.5. (**A**) Representative images of immuno-staining for laminin to identify fetal capillaries. All 7 possible placental types illustrated in Fig. 2A are presented. The HtrA3 genotype of each placental type is schematically shown at the bottom right corner of each image, denoting the maternal decidua basalis genotype in blue on the top and the fetal placental genotype in red on the bottom. The labelling of individual type (a-g) is consistent with the labelling of Fig. 2D. (**B**) Quantitative analysis of laminin staining of the 7 distinctive placentas. Each type (a-g) is labelled consistently between (**A**) and (**B**). Data are expressed as mean ± SD. *p < 0.05, **p < 0.01.

However, the fetal capillary endothelium, detected by immuno-staining for laminin^40^, was distinct from d13 and the differences were profound at d16 (Fig. 3). A small schematic drawing at the bottom right corner of each fetal capillary image in Fig. 3 denotes the HtrA3 genotype of that specific placenta, the genotype of the maternal decidua basalis is shown in blue and that of the fetal placenta in red. The wild type placentas (^+/+^/_+/+_, Fig. 3A-a), without any HtrA3 deletion in either the maternal decidua basalis or the fetal placenta, displayed a well-structured and regularly-spaced fetal capillary network. However, this structure was altered when one HtrA3 allele was deleted only in the maternal decidua basalis and not in the fetal placenta (^+/−^/_+/+_, Fig. 3A-b), the capillaries were not as well organized/connected (Fig. 3A-b vs A-a). This alteration was reflected by a significant reduction in laminin staining intensity (Fig. 3B-b vs B-a). This data indicates that maternal HtrA3 promotes optimal development of the labyrinth fetal capillaries. This is consistent with the wild type E18 fetuses of HtrA3^+/−^ mothers (Fig. 2D-b, supported by ^+/−^/_+/+_ placentas) having lower weight than those of HtrA3^+/+^ mothers (Fig. 2D-a, supported by ^+/+^/_+/+_ placentas).

When one HtrA3 allele was deleted in the fetal placenta but the maternal decidua basalis was wild type (^+/+^/_+/−_, Fig. 3A-c), the fetal capillary appearance was also altered compared to the wild type (^+/+^/_+/+_, Fig. 3A-a), but the disturbance was less severe. This may explain the lack of difference in E18 weight between HtrA3^+/−^ and HtrA3^+/+^ fetuses from wild type mothers (with ^+/+^/_+/−_ and ^+/+^/_+/+_ placentas respectively, Fig. 2D-c and D-a).

The fetal placentas of Fig. 3A-c,d and e were all HtrA3^+/−^, but their maternal decidua basalis was respectively HtrA3^+/+^ (Fig. 3A-c), HtrA3^+/−^ (Fig. 3A-d), and HtrA3^−/−^ (Fig. 3A-e). The fetal capillary networks of 3A-d and 3A-e were more disorganized than that of 3A-c, and their laminin staining intensity was likewise significantly reduced (Fig. 3B-d and B-e vs B-c). This provides further evidence that maternal HtrA3 is important for optimal development of fetal capillaries in the labyrinth. This is consistent with the weight of HtrA3^+/−^ fetuses at E18 being highest when the mother was HtrA3^+/+^ (Fig. 2D-c) and reduced when the mother was HtrA3^+/−^ or ^−/−^ (Fig. 2D-d and D-e).

All placentas with one or both HtrA3 alleles deleted in both maternal and fetal compartments (Fig. 3A-f and A-g), showed abnormal fetal capillaries compared with wild type (Fig. 3A-a), suggesting that the maternal decidual and fetal placental HtrA3 may synergistically influence the labyrinthine fetal capillary development. However, the placenta still developed even when HtrA3 was totally deleted in both components (Fig. 3A-g), suggesting an important supportive rather than a pivotal role of HtrA3 in mouse placentation. These data are in agreement with HtrA3^−/−^ fetuses being viable but with reduced E18 weight when their mothers were HtrA3^+/−^ or HtrA3^−/−^ (Fig. 2D-f and D-g).

### Postnatal growth

**Figure 4:**
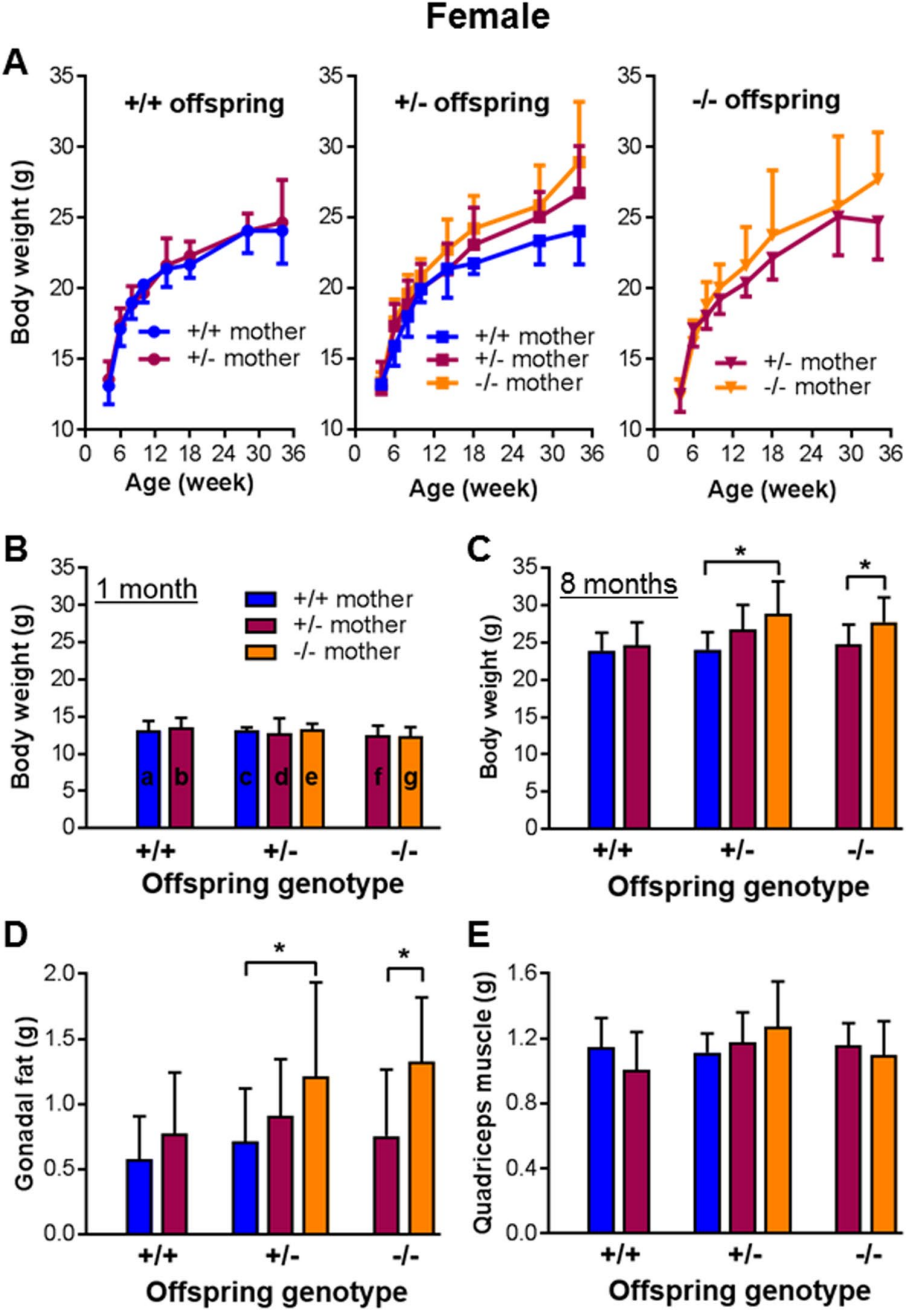
The impact of HtrA3 gene deletion in the mother versus fetus on postnatal body weight and organ weight in the female offspring. Female mice of three HtrA3 genotypes were crossed with HtrA3^+/−^ males as shown in Fig. 2A, and the resulting mice were monitored postnatally. The data from female offspring were analyzed according to their genotype as well as the genotype of their mothers, and are presented in the same format as Fig. 2D. (**A**) Body weight change over the period of 34 weeks. (**B**,**C**) Comparison of body weight across all 7 groups at one (**B**) and eight (**C**) months of age respectively. (**D**,**E**) Weight of gonadal fat (**D**) and quadriceps muscle (**E**) at eight months of age. The numbers of mice examined for each group (similar across **A–E**): a, n = 19; b, n = 9; c, n = 11; d, n = 13; e, n = 11; f, n = 9; g, n = 8. The labelling of a-g is shown on the graph of (**C**) only. Data are expressed as mean ± SD. *p < 0.05.

**Figure 5:**
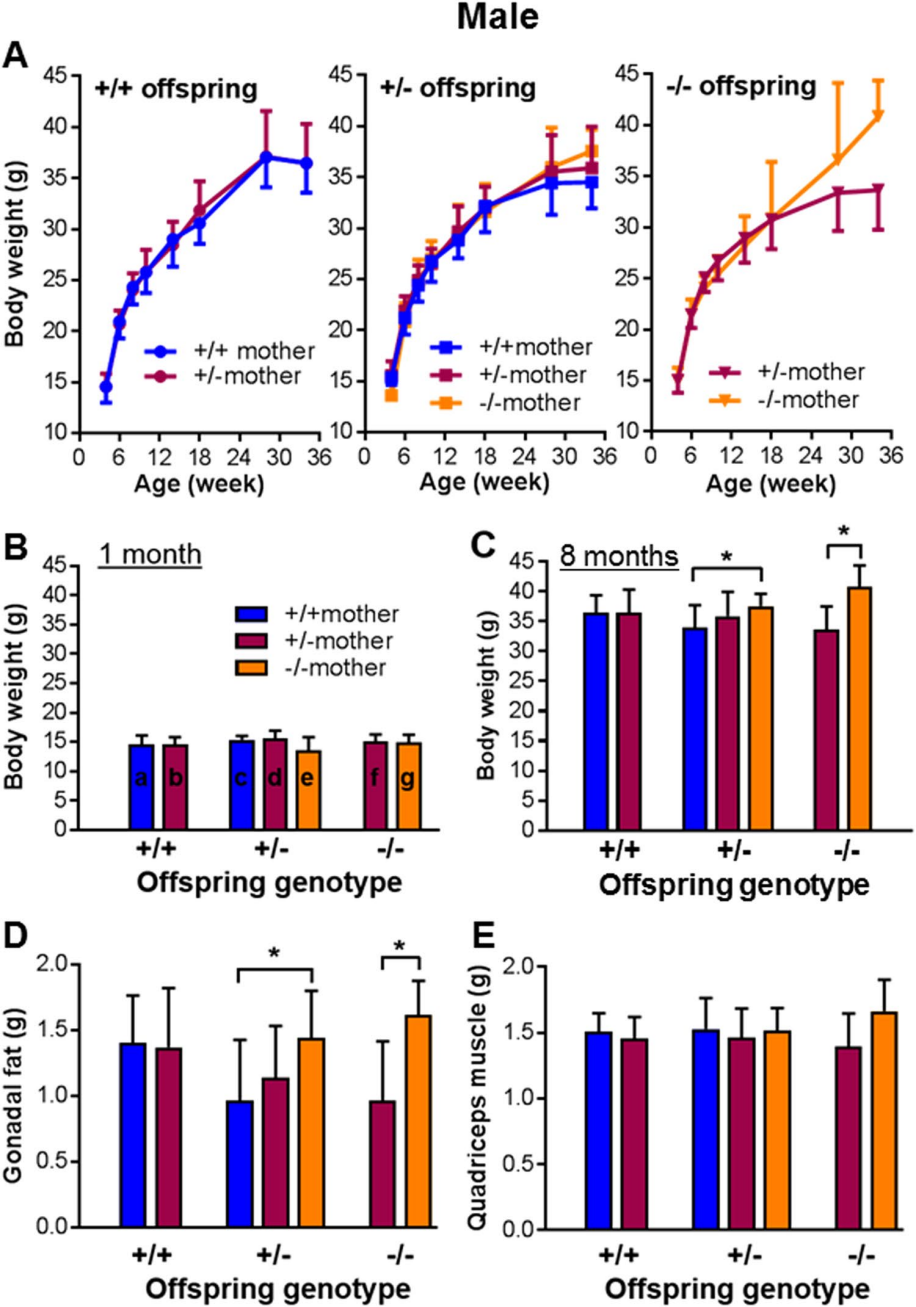
The impact of HtrA3 gene deletion in the mother versus fetus on postnatal body weight and organ weight in the male offspring. Male mice born from the Fig. 2A mating scheme were analyzed according to the genotype of the offspring as well as the genotype of their mothers, and the data are presented in the same format as Fig. 4. (**A**) Body weight change over the period of 34 weeks. (**C**,**D**) Comparison of body weight at one (**B**) and eight (**C**) months of age respectively. (**D**,**E**) Weight of gonadal fat (**D**) and quadriceps muscle (**E**) at eight months of age. The numbers of mice examined for each group (similar across **A**–**E**): a, n = 11; b, n = 13; c, n = 12; d, n = 11; e, n = 11; f, n = 11; g, n = 9. The labelling of a-g is shown on the graph of (**C**) only. Data are expressed as mean ± SD. *p < 0.05.

As IUGR can have long lasting impact on postnatal health, we examined whether the observed mild fetal growth restriction, resulting from suboptimal placentation due to HtrA3 deletion in the mother, would affect the offspring postnatally. Following the breeding scheme of Fig. 2A with extension to natural birth, the postnatal growth of the pups was monitored. Because body weight differs significantly between sexes, females and males were analyzed separately (Figs 4 and 5 respectively). The offspring were again separated into 7 groups according to their own HtrA3 genotype as well as that of their mothers (Figs 4 and 5).

For all female offspring (Fig. 4), body weight was similar at 1 month of age (Fig. 4A,B), but clearly differed over time and at 8 months (mid-adulthood) (Fig. 4A and C). Overall at 8 months of age (Fig. 4C), offspring born to HtrA3^−/−^ mothers were heavier than those of HtrA3^+/+^ mothers, the reverse of their relative fetal weights at E18 (Fig. 2D). Those born to HtrA3^+/−^ mothers showed a non-significant trend to increased weight. There was no difference in weights of wild-type offspring with wild-type or heterozygous mothers. These differences in body weight were likewise observed in male offspring at 8 months of age (Fig. 5A–C).

To further explore this increase in offspring body weight, we analyzed the weight of 10 major organs at 8 months of age. Among gonadal fat pads, visceral fat, brown fat, liver, heart, kidney, spleen, pancreas, quadriceps muscle, and bone (right upper leg), only gonadal fat differed significantly (Figs 4D and 5D). Within each genotype, the gonadal fat was heavier in offspring of HtrA3^+/−^ or HtrA3^−/−^ mothers than wild-type mothers (Figs 4D and 5D), mirroring the pattern of their body weight (Figs 4C and 5C). The weights of other organs examined, including quadriceps muscle (Figs 4E and 5E), were not significantly different. These data thus suggest that the gonadal fat, the largest storage of white fat in the body, is a major contributor to the body weight increase. These results also established an inverse relationship between fetal weight at E18 *in utero* (Fig. 2D), and body and gonadal fat weight in adulthood (Figs 4C,D and 5C,D). Since these comparisons were between mice of similar genotype but with different maternal genotype, it is clear that maternal HtrA3, acting via promotion of optimal placental development, has long-lasting impact on the offspring.

### Serum HtrA3 levels in women during early gestation were lower in IUGR pregnancies

**Figure 6:**
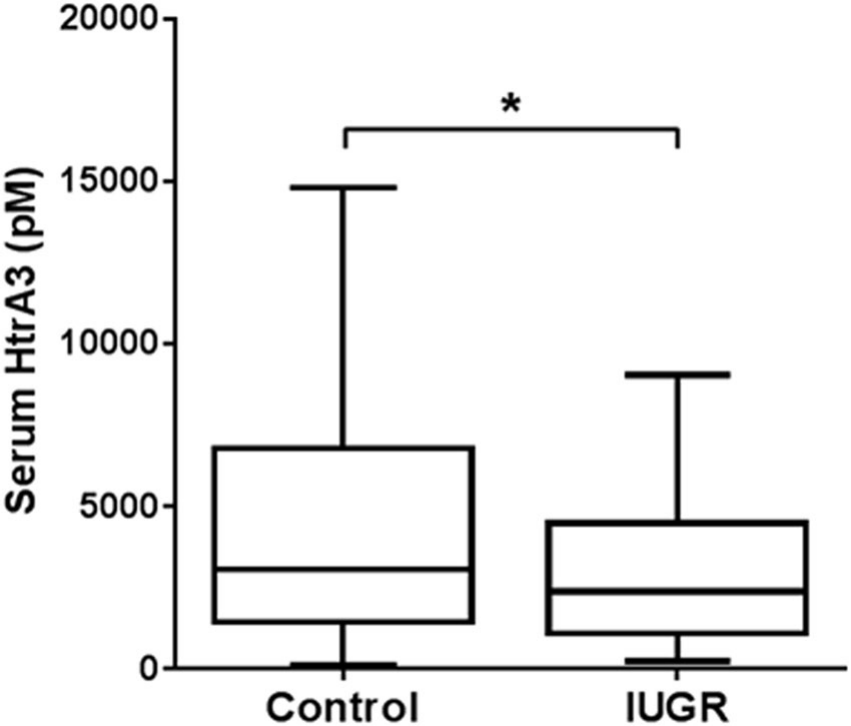
Serum HtrA3 concentration at 11-13 weeks of pregnancy in women who gave birth to normal (Control) or intra-uterine growth restricted (IUGR) babies. An ELISA detecting both HtrA3 isoforms was used to determine serum HtrA3 concentration. Data are presented in Tukey style box and whisker plots. Control, n = 292 (female, 134; male, 158); IUGR, n = 71 (female, 38; male, 33). p = 0.037. Median HtrA3 concentration was significantly lower in IUGR than control pregnancies.

In the human, HtrA3 is highly expressed in the maternal decidual cells and various types of trophoblasts during placental development^25^. In addition, in the human but not in the mouse, HtrA3 is secreted into the maternal circulation^25^. HtrA3 serum concentrations, reflecting decidual and placental production, are highest in the first trimester when the placenta is developing^25^. We thus investigated whether serum HtrA3 concentrations in women during early gestation are different between normal and IUGR pregnancies. We examined a cohort of sera collected at 11–13 weeks of gestation from 363 singleton pregnancies. These women gave birth to normal weight (control, n = 292) or IUGR (<5^th^ percentile, n = 71) babies. Serum HtrA3 levels were significantly lower (p < 0.05) in IUGR than control pregnancies (Fig. 6).

## Discussion

In this study, we investigated the importance of HtrA3 in mouse placentation using a gene deletion strategy. Because a hemochorial placenta requires both fetal and maternal components, and HtrA3 is restricted to the maternal decidual cells in mice, we examined the contribution of maternal vs fetal HtrA3 to placental development. Using a simple but effective mating scheme, a significant supportive role of maternally-derived HtrA3 in optimizing placental development and fetal growth was revealed. The data clearly showed that HtrA3 deletion in the mother affected the organization of the labyrinthine fetal capillaries, which are directly responsible for fetal-maternal exchange. Consistent with this abnormality, deletion of maternal HtrA3 compromised placental function and caused fetal growth restriction irrespective of fetal genotype. Although mild, the IUGR caused by HtrA3 deficiency in the mother significantly affected the growth trajectory of the offspring independent of their own genotype. By adulthood of 8 months of age, mice of identical genotype differed significantly in body weight and white adipose fat content, because of their mothers’ HtrA3 genotype; those born to HtrA3-deficient mothers were significantly heavier with more white fat. Furthermore, in pregnant women, serum levels of HtrA3 during early pregnancy were significantly lower in those who subsequently developed IUGR, establishing an association between lower HtrA3 levels early in pregnancy and placental insufficiency in the human. Collectively these studies demonstrate the importance of HtrA3 in optimizing placental development in mice and humans.

HtrA3 does not appear to be essential for fetal development, as HtrA3^−/−^ mice were born in the expected Mendelian distribution and phenotypically normal. This is also the case for HtrA1 and HtrA4 when they are deleted from the mouse genome^42–44^. However, our results clearly identify that maternal HtrA3 is critical for the development of an optimal and efficient placenta. The major placental defect associated with HtrA3 deletion was disorganization of labyrinthine fetal capillaries. Not surprisingly, this defect resulted in fetal growth restriction because the labyrinthine vasculature is vital to placental function. In mice, the labyrinth comprises finely-branched and interdigitated maternal and fetal blood spaces, which facilitate transfer of nutrients from the mother to the fetus^38, 45^. Consequently, severe abnormalities in this region lead to fetal death, while mild defects cause fetal growth restriction^45–47^. For instance, deletion of HOXA13, PPARγ, HAI-1 or the retinoblastoma gene in mice all results in embryonic lethality because of severe labyrinthine defects^41, 48–50^, whereas lack of placenta-specific IGF-II or p45NF-E2 leads to placental insufficiency and fetal growth restriction^51, 52^.

As all blood spaces within the labyrinth are lined by fetal-derived trophoblasts, the majority of labyrinthine defects reported to date are caused by ablation of genes expressed in these cells^41, 45–52^. In contrast, only a few studies have reported labyrinthine abnormalities following alteration of maternally derived factors. Partial maternal deficiency of maternal Hmox1 is reported to reduce labyrinthine vasculature volume irrespective of fetal genotype^53^. Overexpression of maternal IGFBP-1 results in enlargement of the labyrinth and IUGR, again independent of fetal genotype^54^. Our studies here provide further evidence that maternally-derived factors can influence labyrinthine formation and function. Future studies will investigate how maternal HtrA3 regulates labyrinthine development, including identifying HtrA3 substrates and whether the trophoblasts lining the labyrinthine fetal capillaries express very low levels of HtrA3 that is below the detection limit of the previously used methods of immunohistochemistry and *in situ* hybridization^26, 42^.

Deletion of HtrA1 in mice also results in poor placental development and IUGR^42^. However, the placental defects reported for HtrA1-deficient mice are more severe than in the HtrA3 null mice presented here. The placentas of HtrA1^−/−^ mice are smaller, their junctional zone is reduced and labyrinth vascularization impaired^42^. This is consistent with HtrA1 being expressed in the decidua capsularis in addition to trophoblast precursors^36, 42^, whereas HtrA3 is restricted to maternal decidual cells^26, 42^. In contrast, HtrA4 knockout mice are reported as phenotypically normal and fertile^43^; whether HtrA4 deletion causes subtle placental defects was not reported.

As HtrA3 is highly expressed in maternal decidual cells in both the mouse and human, it was speculated that HtrA3 may be required for the process of stromal decidualization^25, 26^. In this study, decidualization was unaffected in mice deficient in maternal HtrA3, providing strong evidence that HtrA3 is not essential for decidualization at least in mice. However, trophoblast invasion differs considerably between the mouse and human. In mice, endovascular trophoblast invasion is shallow and interstitial trophoblast invasion occurs late in gestation, whereas in the human both endovascular and interstitial invasion is extensive and occurs early during pregnancy^33, 34, 38^. Therefore the role of decidual HtrA3 in human trophoblast invasion needs to be examined in the context of human placentation. In addition, as HtrA3 is expressed in specific trophoblast subtypes such as villous syncytiotrophoblast in the human but not in the mouse, the current mouse study does not shed light on the role of HtrA3 in trophoblast function. However, this study revealed that the maternal HtrA3 serum levels during early pregnancy were significantly lower in women with IUGR than normal pregnancies, implying that HtrA3 may also play an important role in optimizing placental development in the human. Future studies are warranted to establish the causal role of lower HtrA3 in human placental insufficiency. Although preeclampsia (especially the early-onset subtype) and IUGR are often (but not always) linked, HtrA3 serum levels at the end of first trimester are lower in IUGR (this study), but higher in women destined to develop preeclampsia^12^. These differences suggest that HtrA3 is distinctively associated with IUGR and preeclamptic pregnancies.

This study further demonstrated the critical importance of the uterine environment on fetal growth and long-term consequences, consistent with the concept of developmental origins of health and disease (DOHaD)^55–58^. Epidemiological human studies have established that low birth weight is associated with increased rates of adiposity, type 2 diabetes, the metabolic syndrome, and other diseases in adult life^56–59^. This link has also been extensively investigated using animal models^57, 60, 61^. However, most animal studies induce significant fetal growth restrictions through severe maternal undernutrition, maternal uterine artery ligation, maternal ion deficiency, or a low protein diet, which are more appropriate to investigate famine conditions^57, 62^. While these approaches induce placental insufficiency, they are complicated by severe fetal nutrient restriction and significant fetal reprogramming^57^. The present work presents a unique model to investigate the relationship between subtle placental insufficiency, mild IUGR and long-term health consequences, which is more applicable to study the DOHaD phenomenon in the general population.

In addition, our studies present a scenario where adulthood overweight arises from subtle growth restriction *in utero* in the absence of maternal malnutrition or maternal endocrine disturbances. Importantly, such adverse outcomes are independent of an individual’s genetic composition, environmental insults or food access. These results strongly support the theory of the *in utero* origins of obesity^57^, and have important implications in the understanding of the current obesity epidemic.

In summary, the present study revealed the functional importance of HtrA3 in placental development. An effective mating strategy, rather than conditional knockout, was successfully utilized to investigate the contribution of maternal vs fetal HtrA3 to placental development and function. Although HtrA3^−/−^ mice appeared phenotypically normal with undisturbed reproductive function, the maternal HtrA3 played an important role in optimizing placental development and fetal growth. While loss of HtrA3 mildly affected fetal growth *in utero*, it significantly impacted the offspring’s growth trajectory well into adulthood. The association between lower maternal serum levels of HtrA3 early in gestation and IUGR pregnancies in women, suggests that HtrA3 may also play an important role in optimizing human placentation.

## Methods

### Targeted disruption of the HtrA3 gene in mice

A targeting construct (Supplementary Figure 1B), containing two loxP sites, a middle arm (MA) and a flippase recognition target (FRT)-neomycin cassette, was engineered using two BAC (bacterial artificial chromosome) clones spanning the required genomic region of the mouse HtrA3 gene and the ET cloning method^63, 64^. The construct was electroporated into embryonic stem (ES) cells derived from C57BL/6 mice. Exon 1 and the associated sequence were replaced through recombination by the middle arm, the neo cassette and the flanking loxP/FRT sites to produce the targeted allele (Supplementary Figure 1B). The ES cells were subsequently transfected with cre recombinase to remove the middle arm and the neo cassette to create the null allele (Supplementary Figure 1B). Genomic DNA of ES cells was digested with DrdI and SapI separately, and analyzed by standard Southern blot with the 5′ and 3′ probes shown in Supplementary Figure 1B. Correctly targeted ES cells were micro-injected into C57BL/6 J blastocysts and transferred into the uterus of recipient mice, the resulting chimeras were then crossed with C57BL/6 J mice to produce heterozygous offspring, and their tail DNA was analyzed by Southern blot as for the ES cells.

All mice were maintained on a C57BL/6 J background, and they were housed and handled according to the Monash University animal ethics guidelines. All studies were approved by the Animal Ethics Committee at the Monash Medical Centre, Melbourne, Australia.

### Genotyping

Routine genotyping used a PCR strategy shown in Supplementary Figure 1C, with proteinase K digested tail DNA and 55 °C annealing temperature. The primers used were: Forward (F), 5′ACTCTGCTTCCTGGCTACTG3′; Reverse (R) 1, 5′GGAGGTCAAGTTGCTAGTGG3′; and R2, 5′GCCCACCAGACGCTAC3′. PCR products from the very first analysis were confirmed by sequencing. The predicted PCR products of different genotypes are shown in the Table of Supplementary Figure 1C.

### Confirmation of HtrA3 deletion at the mRNA and protein levels

Deletion of HtrA3 mRNA was confirmed by Northern blot analysis of total RNA from d10.5 implantation sites that contained the maternal decidua basalis as well as the fetal placenta as previously published^26^. The morning of finding a vaginal plug was designated as day 0 of pregnancy. Northern analysis utilized four distinct probes as illustrated in Supplementary Figure 1A, to detect different regions of the two HtrA3 mRNA variants. Each blot was also probed with a mouse cDNA for 18 S ribosomal RNA. HtrA3 proteins was confirmed by standard Western blot analysis of total proteins of d10.5 implantation sites as previously reported^26^. HtrA3 deletion was also confirmed by immunohistochemistry as published^26^. An HtrA3 antibody that was previously confirmed to detect both HtrA3 isoforms^26^, was used for both Western blot and immunohistochemical analyses.

### Mouse breeding and analysis

To determine if HtrA3 deletion is detrimental to reproduction, female mice of three HtrA3 genotypes (+/+, +/− and −/−) were mated with the same three genotypes of male mice, and the resulting pups were counted and examined for gross abnormalities at birth and at weaning (4 weeks of age). To determine the contribution of maternal vs fetal HtrA3 to placental development and E18 fetal weight, the breeding strategy illustrated in Fig. 2A was utilized. Fetal sex at E18 was determined by PCR of Y chromosome specific gene SRY as previously reported^65^. The same breeding scheme was utilized to analyze the impact of HtrA3 deletion in the mother vs fetus on offspring’s postnatal growth. The pups were born naturally, and their body weight was monitored monthly from weaning till 34 weeks (~8 months) of age. At the end of the experiment, 10 major organs (gonadal fat pads, visceral fat, brown fat, liver, heart, kidney, spleen, pancreas, quadriceps muscle, and bone from the right upper leg) were carefully dissected and immediately weighed.

### Histological analysis

Intact implantation sites containing the uterine, placental and fetal tissues were fixed in 10% neutral buffered formalin (pH 7.6) and processed as previously described^26^. All analyses employed 5-micron sections, and all comparisons were made using mid-sagittal sections. All immuno-staining, except for Factor VIII, required antigen retrieval by microwaving the sections in 0.01 M citrate buffer for 10 min, and all were counter-stained with Harris’ hematoxylin (1:10) and mounted with DPX (BDH Chemicals, Kilsyth, Victoria, Australia).

The maternal decidual cells were immuno-stained for desmin^26^, the decidual NK cells were stained with lectin-DBA^26, 37^. The decidual endothelium was immuno-stained for Factor VIII^38^. The glycogen cells within the junctional zone were stained with periodic acid–schiff (PAS), followed by digestion without and with α-amylase as reported^38^. Junctional zone glycogen cells were also immuno-stained for CX31^39^. Glycogen cells in the maternal decidua were immuno-stained for decorin^39^. The alkaline phosphatase activity in the labyrinth was determined to analyze the maternal blood sinuses^40^. Maternal blood sinuses were also analyzed by isolectin BS1 binding as published^41^. The fetal capillaries in the labyrinth were detected by immuno-staining for laminin^40^, using a rabbit anti-mouse laminin polyclonal antibody (Sigma Aldrich) and biotinylated goat anti-rabbit IgG (Vector laboratories) as the secondary antibody. To quantify laminin staining, images at 60x magnification were taken from 5 different areas within each section, and the images were analysed by the Fiji open-source platform^66^. A total of 15 areas from 3 different placentas were analysed for each unique placental type.

### Analysis of HtrA3 in sera from pregnant women

An ELISA proven to detect both isoforms of HtrA3 in human serum was employed to determine HtrA3 concentrations during early pregnancy. The cohort was collected at 11–13 weeks of pregnancy at Royal Prince Alfred Hospital, Sydney, Australia; the participants were originally recruited to a study evaluating an algorithm for predicting pregnancy diseases in the first trimester^67, 68^. For the current study, singleton pregnant sera from pregnancies that gave birth to normal weight (control) and IUGR (<5^th^ percentile) babies were randomly selected from the above collection. Control, n = 292 (female, 134; male, 158). IUGR, n = 71 (female, 38; male, 33). The study was conducted in accordance with ethics approval from Royal Prince Alfred Hospital, Sydney, Australia (Study No. X11–0305 & HREC/11/RPAH/472).

### Statistics

All statistical analysis used PRISM version 6.00 (GraphPad Software, San Diego, CA), and two-tail p < 0.05 was taken as significant. For the mouse studies, unpaired t-test (for 2 groups) or one-way ANOVA (3 groups) were performed, and data expressed as mean ± SD. For the human studies, as the data was not normally distributed, Mann-Whitney analysis was used and the results were presented in Tukey style box and whisker plots.

## Electronic supplementary material

Supplementary information
