# Supplementary material for: Maternal HtrA3 optimizes placental development to influence offspring birth weight and subsequent white fat gain in adulthood
Source: Sci Rep. 2017 Jul 4;7:4627. doi: 10.1038/s41598-017-04867-3 (PMC5496872; doi:10.1038/s41598-017-04867-3)
Supplement: Supplementary file 1 — Supplementary information [file 41598_2017_4867_MOESM1_ESM.pdf]

Maternal HtrA3 optimizes placental development to influence offspring birth weight and subsequent white fat gain in adulthood

Ying Li[1,2], Lois A. Salamonsen[1,2,3], Jonathan Hyett[4,5], Fabricio da Silva Costa[3,6] and Guiying Nie[1,2,7*]

[1]Centre for Reproductive Health, Hudson Institute of Medical Research, Clayton, Victoria, Australia

[2]Department of Molecular and Translational Sciences, [3]Department of Obstetrics and Gynaecology, Monash University, Clayton, Victoria, Australia

[4]Central Clinical School, University of Sydney, Sydney, New South Wales, Australia

[5]RPA Women and Babies, Royal Prince Alfred Hospital, Sydney, New South Wales, Australia

[6]Monash Ultrasound for Women, Melbourne, Victoria, Australia

[7]Department of Biochemistry and Molecular Biology, Monash University, Clayton, Victoria, Australia

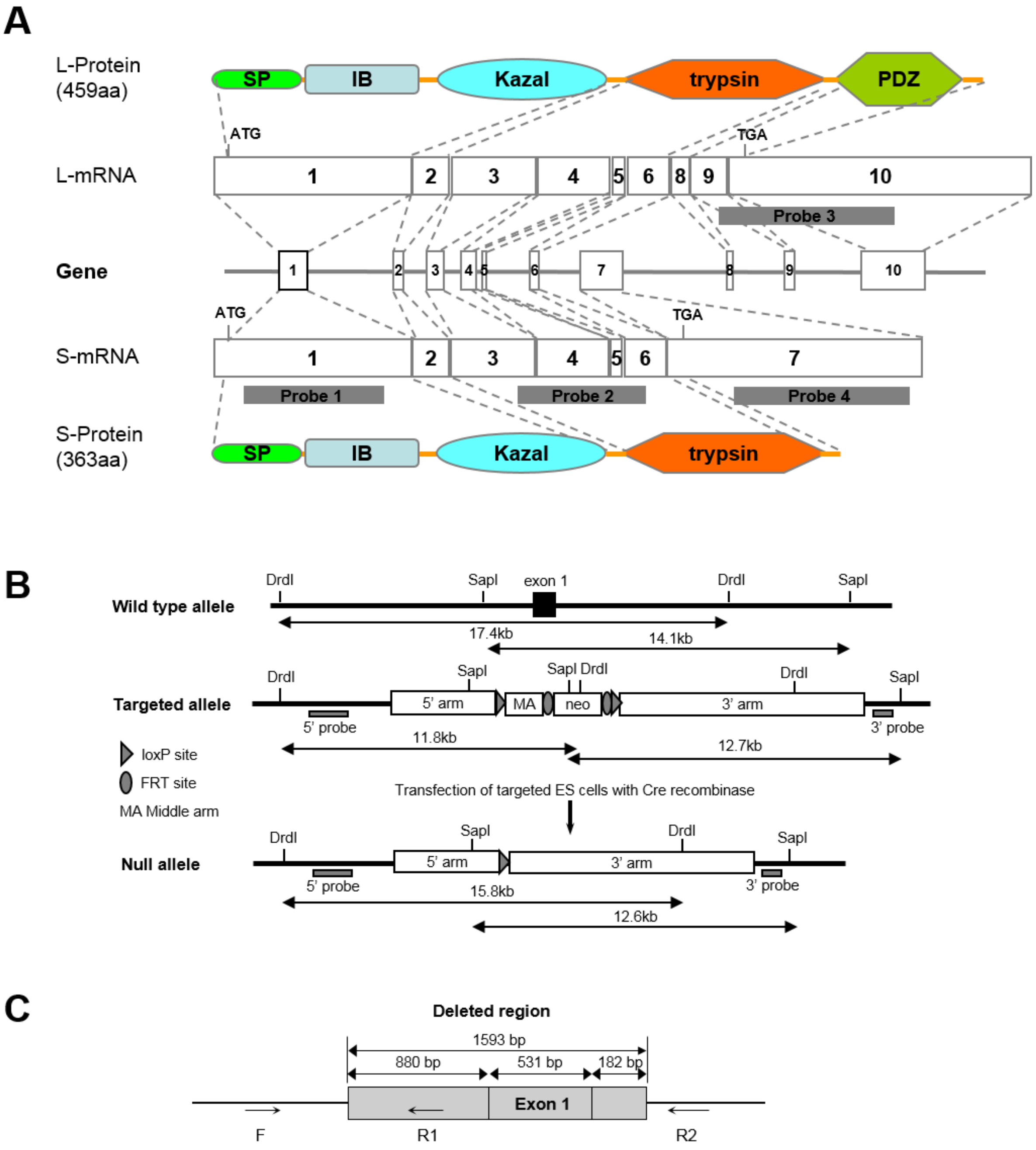

PCR products

Primers	+/+	+/-	-/-
F + R1	374 bp	374 bp	N/A (missing)
F + R2	N/A (too big)	271 bp	271 bp

Supplementary Figure 1

Supplementary Figure 1: Targeting strategy for deletion of the HtrA3 gene in the mouse. (A) The mouse HtrA3 gene intro-exon structure, the two alternatively spliced HtrA3 mRNA variants (L-mRNA and S-mRNA), and the two resulting HtrA3 protein isoforms (L-Protein and S-Protein). Exons are illustrated, in relative size proportion, by boxes and sequentially numbered on the gene and the mRNAs; introns are represented by the gaps between the exons. On the mRNAs, the translation start codon ATG on exon 1, and the stop codon TGA on exons 7 (S-mRNA) and 10 (L-mRNA), are also shown. The SP (signal peptide), IGFB (IGF-binding), Kazal (Kazal-type S protease inhibitor) and trypsin (trypsin-like serine protease) domains are shared between the two HtrA3 protein isoforms (L-protein, 459aa; S-Protein, 363aa), whereas the C-terminal PDZ domain is present only in the L-Protein isoform. The contribution of individual exons to the translated protein domains is illustrated. The locations of 4 probes for Northern blot analysis are also shown. Probe 1, specific to exon 1 and common to both HtrA3 mRNAs; probe 2, down-stream of exon 1 and shared between the two HtrA3 mRNAs; probe 3, specific to HtrA3 L-mRNA; probe-4, unique to HtrA3 S-mRNA. **(B) Targeting strategy to delete exon 1 and its flanking sequences in the mouse HtrA3 gene**. Top, the wild type gene allele surrounding exon 1 and the DrdI and SapI restriction sites. Middle, the targeted allele in which exon 1 and its flanking sequences are replaced by the middle arm (MA), the neomycin cassette (neo) and the flanking FRT and loxP sites. Also shown are the 5' and 3' arms for homologous recombination, and the two Southern blot probes (5' probe for DNA digested with DrdI, 3' probe for DNA digested with SapI). Bottom, the null allele resulting from transfection of the targeted ES cells with Cre recombinase to remove MA and neo cassette. **(C) Details of the deleted region and the PCR genotyping strategy**. The deleted region (1593 bp) comprises the entire exon 1 (531 bp), plus 880 bp preceding and 182 bp following exon 1. One forward (F) and two reverse (R1 and R2) genotyping primers are shown, F and R2 are outside whereas R1 is within the deleted region. The predicted PCR

products with these 3 primers (F paired with R1 and R2 respectively) for the wild (+/+, 374 bp), heterozygous (+/-, 374 bp and 271 bp) and homozygous (-/-, 271 bp) alleles are summarized in the Table below. N/A, not amplifiable.
